# Factors Associated with Caregiving and Mental Stress among Family Caregivers for People with Disabilities: A Cross-sectional Survey of 40,600 Chinese Households

**DOI:** 10.1007/s11524-026-01111-0

**Published:** 2026-07-27

**Authors:** Xingnan Yi, Zhaohua Huo, Xuechen Xiong, Karen Ann Grépin, Tarani Chandola, Wai Sze Chan, Vera Mun Yu Tang, Vivian Weiqun Lou, Jianchao Quan

**Affiliations:** 1 School of Public Health, The University of Hong Kong, Pokfulam, Hong Kong; 2 Department of Psychiatry, Faculty of Medicine, The Chinese University of Hong Kong, Sha Tin, New Territories Hong Kong; 3 Department of Applied Social Sciences, The Hong Kong Polytechnic University, Hung Hom, Hong Kong; 4 Department of Sociology, The University of Hong Kong, Pokfulam, Hong Kong; 5 Department of Psychology, The University of Hong Kong, Pokfulam, Hong Kong; 6 Department of Social Work and Social Administration, The University of Hong Kong, Pokfulam, Hong Kong; 7 Sau Po Centre On Ageing, The University of Hong Kong, Pokfulam, Hong Kong

**Keywords:** Informal care, Family caregiving, Disabilities, Mental health, East Asia

## Abstract

Family caregivers in East Asian societies such as Hong Kong bear a significant share of care responsibilities for individuals with disabilities due to rising disability prevalence, limited formal services, and cultural norms rooted in filial piety. This study examines factors associated with caregiving provision, weekly caregiving hours, and mental stress among family caregivers in Hong Kong households. We used data from the 2019–2020 Hong Kong General Household Survey (*n* = 40,600) and applied logistic regression (caregiving provision) and ordinal logistic regression (caregiving hours, mental stress). Analyses were adjusted for care recipient needs, caregiver characteristics, and contextual factors. Disability types were classified using the International Classification of Functioning, Disability and Health (ICF) framework. Among weighted respondents from households including individuals with disabilities in Hong Kong, 14.5% were identified as family caregivers. Caregiving was significantly associated with being female (adjusted odds ratio [aOR] = 1.64, 95% CI = 1.49–1.82) and unmarried (aOR = 1.17, 95% CI = 1.02–1.34). Female (aOR = 1.33, 95% CI = 1.12–1.59) and assisting with activities of daily living (ADLs) were associated with longer weekly caregiving hours (aOR = 2.54, 95% CI = 2.12–3.04). Severe self-reported mental stress was associated with female (aOR = 1.39, 95% CI = 1.15–1.69), assisting with ADLs (aOR = 1.99, 95% CI = 1.62–2.44), and caring for family members with mental health disability (aOR = 2.00, 95% CI = 1.64–2.43). While other ICF-based disability types were not significant in multivariate models, some showed associations in univariate analysis. These findings highlight the need for targeted caregiver support policies that address gendered expectations and disability-specific burdens.

## Introduction

Around 16% of the global population (1.3 billion people) have significant disabilities, which include physical, intellectual, sensory, and mental illness [1]. Much of the caregiving for these individuals falls to unpaid family members, who provide essential support such as help with daily activities, medication management, emotional support, and navigating services [2]. In East Asia, caregiving roles are further intensified by the influence of Confucian cultural norms such as filial piety (*Xiao*), which emphasize intergenerational obligations [3, 4]. Hong Kong exemplifies this intersection of demographic change, cultural expectation, and service limitations. Over the past decade, the disability prevalence in Hong Kong has surged by 36.5%, rising from 5.2% in 2007 to 7.1% in 2020 [5]. However, formal long-term care infrastructure has not kept pace. Waiting lists for both residential and community-based services can extend for years, placing most of the burden on families [6].

Critical gaps persist in characterizing caregiving burdens faced by disability caregivers, particularly in East Asian contexts where homogenized research approaches obscure heterogeneous caregiving experiences. In many empirical studies, disability is operationalized as a single binary indicator or examined within narrowly defined diagnostic groups, leading to an implicit assumption that caregiving demands are broadly comparable across conditions and thereby obscuring important heterogeneity in caregiving experiences [7, 8]. Caregiver burden involves not just the likelihood of providing care, but also its intensity and psychological effects. While studies identify individual, relational, and contextual predictors of caregiver burden, such as the caregiver’s gender, employment status, and available support, they frequently treat “disability” as a uniform category, neglecting how distinct conditions generate divergent demands [9–13]. For example, supporting someone with physical limitations such as bathing or feeding can involve intense physical labor and time [14]. In contrast, caregivers supporting individuals with mental health conditions or social participation restrictions may encounter unpredictable behavioral challenges, stigma, and emotional fatigue [15, 16]. This methodological gap mirrors policy neglect: Japan’s Long-Term Care Insurance excludes working-age disabled adults, and Hong Kong’s 2023 caregiver subsidies target only eldercare [17, 18].

To address these gaps, this study integrates the WHO’s International Classification of Functioning, Disability and Health (ICF) with Broese van Groenou and De Boer’s caregiving model, creating a multidimensional lens for analyzing burden disparities. The ICF provides a comprehensive definition of disability across several domains: limitations in body function and structure, and restrictions in activity and participation [19]. Applying this framework reveals important differences in caregiver demands. This study applies the ICF framework to examine caregiving across a broader spectrum of disability types, allowing for a more comprehensive understanding of how care needs shape both time burden and mental stress. To guide our analysis and variable selection, we adopt the informal care model by Broese van Groenou and De Boer [20], which conceptualizes caregiving as a function of three interconnected domains: the care recipient’s needs, the caregiver’s individual characteristics, and the broader social and support environment. This model complements the ICF framework by emphasizing the relational and contextual dimensions that shape how caregiving is distributed and sustained over time.

This study aims to examine factors associated with caregiving provision, intensity, and mental stress among family caregivers of individuals with disabilities in Hong Kong, leveraging population-representative data to inform equitable support frameworks. By focusing on this often-neglected population, we seek to address the evidence gap surrounding caregiving for people with disabilities in high-density, aging societies where informal care is dominant. Our contributions are twofold. First, we apply the ICF framework to capture the diversity of disability-related care demands, examining how specific types of disability are associated with caregiver outcomes. Second, we explore how individual, relational, and contextual characteristics shape who provides care, how much care is provided, and what stress results from it. Using population-representative data from the Hong Kong General Household Survey (*n* = 40,600), this study offers new insights into how caregiving burdens are shaped in dense, aging urban societies, with implications for future public health and social care planning in East Asia and beyond.

## Methods

### Data Source

We derived study data from the General Household Survey (GHS) conducted by the Census and Statistics Department (C&SD) of Hong Kong between August 2019 and December 2020. The GHS sampling frame included all land-based residential units in Hong Kong, covering both urban or other major developed areas (“built-up”) areas and rural or less densely developed (“non-built-up”) areas. These units comprise conventional housing (e.g., flats and houses) as well as premises with mixed residential use. Sampling was conducted using two complementary frames: the Register of Quarters, which covers housing units in built-up areas, and the Register of Segments, which consists of geographically defined clusters of dwellings in non-built-up areas (e.g., village houses or temporary structures). For the survey on persons with disabilities, 40,600 households were successfully enumerated, representing approximately 1.5% of all households in Hong Kong, with a response rate of 72%.

We utilized GHS data weighted by age, sex, district, and socioeconomic status as provided by the C&SD. The weighted dataset represented approximately 1,017,700 individuals living in households that included at least one person with a disability in Hong Kong. Information on caregiving was collected in relation to these individuals with disabilities. Respondents were asked whether they received unpaid care, and where multiple caregivers were involved, the primary caregiver was defined as the individual providing the greatest number of caregiving hours per week. After applying the inclusion criterion of providing unpaid care at least once per week, the analytic sample included approximately 148,000 family caregivers and 869,700 non-caregivers among individuals living in households that included persons with disabilities. Non-caregivers (i.e., individuals not providing care within these households) were retained as a comparison group to assess factors associated with caregiving provision.

### Variables

#### Outcomes

The dependent variables in this study were (1) caregiving provision, (2) weekly caregiving hours, and (3) mental stress severity reported by family caregivers.

Caregiving provision was assessed by asking respondents whether they had provided unpaid healthcare, personal care, or other forms of assistance to at least one household member with disabilities at least once per week. Respondents who answered “yes” were then asked to report their weekly caregiving hours and their level of mental stress related to caregiving. Weekly caregiving hours were recorded as a categorical variable, divided into six ranges: “0–20 hours,” “20–40 hours,” “40–60 hours,” “60–80 hours,” “80–100 hours,” and “more than 100 hours” per week. Mental stress severity was measured through self-report and categorized as “No stress,” “A little stress,” “Moderate stress,” and “A lot of stress”.

#### Independent Variables

Guided by the informal care model developed by Broese van Groenou and De Boer [20], independent variables were grouped into three domains: care recipient needs, caregiver characteristics, and contextual factors.

Care recipient needs included the type of disability, specific care tasks required, age, and sex of the care recipient. Disability types were classified according to the ICF framework, which conceptualizes disability across two broad domains: limitations in body function and structure, and restrictions in activity and participation. Following this framework, impairments included seeing difficulties, hearing difficulties, restricted body movement, and communication difficulties, as self-reported by respondents experiencing significant difficulties lasting or expected to last 6 months or more. Additionally, mental health conditions and neurodevelopmental disorders—including mental illness/mood disorders, autism spectrum disorder (ASD), specific learning disabilities (SpLD), and attention deficit/hyperactivity disorder (AD/HD)—were included as professionally diagnosed impairments within the past 12 months. Care tasks were categorized into activities of daily living (ADLs), such as dressing, bathing, toileting, feeding, and transferring; and instrumental activities of daily living (IADLs), including meal preparation, healthcare coordination, communication, and going out.

Family caregiver characteristics included sex, age, marital status, education level, and employment status. Finally, contextual factors were assessed through the availability of additional caregiving support from other family members or co-resident caregivers (i.e., individuals living in the same household who also provide care, such as live-in helpers).

### Statistical Analysis

We applied descriptive statistics to compare the characteristics of caregivers and non-caregivers, based on population weighting. Differences in characteristics between caregivers and non-caregivers were assessed using chi-square tests. We used logistic regression to examine factors influencing caregiving provision, and ordinal logistic regression to analyze weekly caregiving hours and mental stress severity. For categorical variables with more than two categories, reference groups were selected based on sample size and their position within the distribution, typically using groups with a relatively large number of observations and representing central categories to facilitate comparisons with both lower and higher groups. Variables with a *p*-value < 0.25 in univariate analyses were included in the multivariable regression models. The threshold of *p* < 0.25 indicated some reasonable association with the outcome [21]. We calculated adjusted odds ratios (aOR) with 95% confidence intervals (CI). Variance inflation factor (VIF) tests were conducted to assess multicollinearity among variables. To minimize Type I errors due to multiple comparisons, false discovery rate (FDR)–adjusted *p*-values were applied. Analyses were conducted using R (Version 4.2.2).

## Results

### Characteristics of Family Caregivers and Non-caregivers

Table 1 presents the characteristics of individuals living in households that included at least one person with a disability. Among the weighted population, 14.5% (*n* = 148,000) were family caregivers. Compared to non-caregivers, caregivers included a higher proportion of females and individuals aged 30–49 and 50–69, while non-caregivers had a larger proportion aged 70 and above (all *p* < 0.001). Caregivers had a higher proportion of individuals who were currently married, whereas non-caregivers included a greater share of widowed, divorced, or separated individuals. In addition, caregivers had higher levels of educational attainment and a higher proportion of employed individuals.

**Table 1:** Weighted characteristics of individuals living in households with persons with disabilities, by caregiving status

Variables	Family caregivers (*n* = 148,000)		Non-caregivers (*n* = 869,700)		*p*-value
	*N*	%	*N*	%	
**Female**	97,200	65.6	491,600	56.5	< 0.001
**Age**					
< 15	< 250	0.1	24,200	2.8	< 0.001
15–29	5300	3.6	46,500	5.3	
30–49	48,600	32.8	122,500	14.1	
50–69	69,000	46.6	367,300	42.2	
> = 70	25,100	17.0	309,300	35.6	
**Employed**	61,200	41.3	247,300	28.4	< 0.001
**Marital status**					
Never married	37,400	25.2	156,900	18.0	< 0.001
Currently married	96,800	65.3	490,300	56.5	
Widowed/divorced/separated	13,900	9.5	222,500	25.5	
**Educational attainment**					
Below primary	33,000	22.3	344,700	39.6	< 0.001
Lower/upper secondary	76,300	51.5	374,600	43.1	
Post-secondary	38,700	26.2	150,400	17.3	
**Relationship with care recipient**					
Spouse	45,800	30.9	N.A		
Parent	35,600	24.0	N.A		
Children/children-in-law	59,600	40.3	N. A		
Sibling/other relative	6500	4.4	N.A		
Others	500	0.4	N.A		
**Female care recipient**	78,000	52.7	N.A		
**Age of care recipient**					
< 15	24,000	16.2	N. A		
15–29	9500	6.4	N.A		
30–49	8900	6.0	N.A		
50–69	24,300	16.4	N.A		
> = 70	81,400	55.0	N.A		
**If have extra help from other caregivers**					
Yes	62,600	42.3	N.A		
**Caring activity needed to provide**					
Activities of daily living (ADLs)	60,200	40.6	N.A		
Instrumental activities of daily living (IADLs)	129,900	87.7	N.A		
**Type of care recipient’s disability (ICF-based)**					
Seeing disability	16,100	10.9	N.A		
Hearing disability	15,300	10.4	N.A		
Communication disability	16,100	10.8	N.A		
Restriction in body movement	87,800	59.3	N.A		
Specific learning difficulties (SpLD)	14,500	9.8	N.A		
Attention deficit/hyperactivity disorder (AD/HD)	16,200	10.9	N.A		
Autism spectrum disorder (ASD)	12,600	8.5	N.A		
Mental illness/mood disorder	45,700	30.8	N.A		
**Hours of care per week**	39.7	(38.5, 41.1)			
0– < 20	38,700	26.2	N.A		
20– < 40	49,900	33.7	N.A		
40– < 60	29,000	19.6	N.A		
60– < 80	11,400	7.6	N.A		
80– < 100	9900	6.7	N.A		
> = 100	9200	6.2	N.A		
**Mental stress severity**^**†**^					
No stress	60,300	47.0	N.A		
A little stress	41,200	32.1	N.A		
Moderate stress	21,100	16.4	N.A		
A lot of stress	5800	4.5	N.A		

^**†**^13.3% of the responses reported “did not know” or refused to answer

a) Non-caregivers refer to individuals living in households with persons with disabilities who did not provide caregiving

b) For those who had specific needs and had more than one person taking care of their day-to-day living, caregiver in the table refers to the one who provided the longest hours of caring services during a week

c) All values are weighted and rounded up to the nearest hundred to account for survey design

Among caregivers, most were female (65.6%), aged between 50 and 69 years (46.6%), unemployed (58.7%), married (65.3%), and had attained at least secondary education (51.5%). Primary caregivers were typically the children or children-in-law of the recipient (40.3%), and most provided care independently without additional help from other family members or caregivers (57.7%). Care recipients spanned all age groups, with the largest proportion aged 70 years or older (55.0%). Nearly all caregivers (87.7%) were responsible for IADL tasks, primarily assisting with care recipients who had restrictions in body movement (59.3%). The mean weekly caregiving hours provided by caregivers was 39.7 h, and 53.0% of them reported experiencing mental stress due to their caregiving responsibilities.

### Factors Associated with Provision of Family Care

Significant factors associated with providing family caregiving for individuals with disabilities included sex, age, marital status, and educational attainment of respondents (Table 2). Female caregivers were more likely to provide family care compared to male caregivers (aOR = 1.64, 95% CI = 1.49–1.82). Individuals aged 30–49 were more likely to provide caregiving compared to those aged 50–69 (aOR = 1.86, 95% CI = 1.63–2.12), while those age < 30 or age ≥ 70 were less likely to provide caregiving. Never married individuals were more likely to provide care compared to married individuals (aOR = 1.17, 95% CI = 1.02–1.34). Conversely, widowed, divorced, or separated individuals were less likely to give care compared to married individuals (aOR = 0.32, 95% CI = 0.27–0.38). Caregivers with less than primary education were less likely to provide care compared to those with secondary education (aOR = 0.70, 95% CI = 0.62–0.80).

**Table 2:** Logistic regression analysis of factors associated with family caregiving provision for people with disabilities

Variables	Crude OR (95% CI)	Adjusted OR (95% CI)^†^
**Sex**		
Female	Ref	Ref
Male	0.95 (0.94, 0.97)	0.61 (0.55, 0.67)***
**Age**		
< 15	0.86 (0.85, 0.87)	0.02 (0.01, 0.07)**
15–29	0.95 (0.92, 0.97)	0.49 (0.37, 0.64)***
30–49	1.13 (1.11, 1.16)	1.86 (1.63, 2.12)***
50–69	Ref	Ref
> = 70	0.92 (0.91, 0.93)	0.59 (0.51, 0.68)***
**Employment status**		
Employed	1.08 (1.06, 1.09)	1.08 (0.96, 1.21)
Unemployed	Ref	Ref
**Marital status**		
Never married	1.03 (1.01, 1.05)	1.17 (1.02, 1.34)*
Currently married	Ref	Ref
Widowed/divorced/separated	0.90 (0.89, 0.91)	0.32 (0.27, 0.38)***
**Educational attainment**		
Below primary	0.92 (0.91, 0.93)	0.70 (0.62, 0.80)***
Lower/upper secondary	Ref	Ref
Post-secondary	1.04 (1.02, 1.05)	1.09 (0.96, 1.24)

***p <.001, **p <.01, *p <.05, *p*-values are adjusted for multiple comparisons using the Benjamini-Hochberg (FDR) procedure; *n* = 148,000

^**†**^Adjusted odds ratios (aOR) are derived from multivariate ordinal logistic regression analyses, where only variables with a *p*-value < 0.25 in univariate analysis were included in the multivariate model

### Factors Associated with Weekly Caring Hours Provided by Family Caregivers

Factors associated with weekly caregiving hours provided by family caregivers and mental stress experienced by family caregivers due to caregiving responsibilities are shown in Table 3 (statistically significant results only; full regression results in Appendix Table 4). Significant factors influencing the amount of weekly caregiving hours included the care recipients’ required activities, caregivers’ sex, employment status and educational attainment, and the availability of extra help from other caregivers or family members. Caregivers assisting those who required help with ADLs were more likely to provide longer weekly caregiving hours compared to caregivers assisting recipients who did not require help with ADLs (aOR = 2.54, 95% CI = 2.12–3.04). Female caregivers were more likely to provide longer caregiving hours compared to male caregivers (aOR = 1.33, 95% CI = 1.12–1.59). Employed caregivers were less likely to provide extensive caregiving compared to unemployed caregivers (aOR = 0.35, 95% CI = 0.29–0.41). Caregivers with post-secondary education were less likely to provide longer caregiving hours compared to those with secondary education (aOR = 0.62, 95% CI = 0.51–0.75). Caregivers with extra help from other caregivers or family members were less likely to spend longer weekly caregiving hours compared to those without any help (aOR = 0.55, 95% CI = 0.47–0.64).

**Table 3:** Significant results from logistic regression analysis of associations between care recipients’ needs, family caregivers’ characteristics, contextual factor, and weekly caregiving hours provided by caregivers and mental stress severity experience by family caregiver due to caregiving responsibilities for people with disabilities

Variables	Weekly caregiving hours provided		Level of mental stress felt	
	Crude OR (95% CI)	Adjusted OR (95% CI)^†^	Crude OR (95% CI)	Adjusted OR (95% CI)^†^
**Age of care recipient (ref: 50–69)**				
> = 70	1.02 (0.84, 1.25)	0.85 (0.67, 1.09)	0.73 (0.58, 0.91)	0.60 (0.56, 0.80)**
**Caring activity required**				
Activities of daily living (ADLs) (ref: do not need ADLs)	2.00 (1.73, 2.32)	2.54 (2.12, 3.04)***	1.54 (1.31, 1.81)	1.99 (1.62, 2.44)***
**Type of care recipient’s disability (ICF-based)**				
Seeing disability (ref: no seeing disability)	0.84 (0.67, 1.06)	0.91 (0.72, 1.16)	0.69 (0.52, 0.90)	0.83 (0.62, 1.12)
Hearing disability (ref: no hearing disability)	0.95 (0.75, 1.20)	-	0.95 (0.73, 1.23)	-
Communication disability (ref: no communication disability)	1.69 (1.33, 2.14)	1.34 (1.04, 1.72)	1.64 (1.27, 2.11)	1.21 (0.92, 1.59)
Restriction in body movement (ref: no restriction)	1.17 (1.01, 1.35)	1.09 (0.89, 1.35)	0.81 (0.69, 0.95)	1.39 (1.07, 1.80)
SpLD (ref: no SpLD)	1.38 (1.09, 1.76)	1.14 (0.85, 1.53)	1.62 (1.24, 2.12)	0.83 (0.60, 1.15)
AD/HD (ref: no AD/HD)	1.08 (0.86, 1.35)	-	2.12 (1.63, 2.76)	1.24 (0.87, 1.76)
ASD (ref: no ASD)	1.07 (0.83, 1.38)	-	1.92 (1.45, 2.54)	1.06 (0.74, 1.51)
Mental illness/mood disorder (ref: no mental illness)	1.02 (0.87, 1.19)	-	1.72 (1.45, 2.05)	2.00 (1.64, 2.43)***
**Female caregiver (ref: male caregiver)**	1.72 (1.49, 2.00)	1.33 (1.12, 1.59)**	1.67 (1.41, 1.96)	1.39 (1.15, 1.69)**
**Employment status of caregiver (ref: unemployed)**				
Employed	0.25 (0.21, 0.29)	0.35 (0.29, 0.41)***	0.83 (0.71, 0.98)	0.97 (0.80, 1.17)
**Educational attainment of caregiver (ref: lower/upper secondary)**				
Post-secondary	0.45 (0.37, 0.53)	0.62 (0.51, 0.75)***	0.80 (0.66, 0.97)	0.88 (0.71, 1.09)
**Have extra help (ref: no extra help)**	0.54 (0.47, 0.63)	0.55 (0.47, 0.64)***	0.94 (0.80, 1.11)	-

***p <.001, **p <.01, *p <.05, *p*-values are adjusted for multiple comparisons using the Benjamini-Hochberg (FDR) procedure; *n* = 148,000

^†^Adjusted odds ratios (aOR) are derived from multivariate ordinal logistic regression analyses, where only variables with a *p*-value < 0.25 in univariate analysis were included in the multivariate model

“-” indicates variables not entered into the multivariable model. Variables with *p* ≥ 0.25 in univariate analyses were not included in the multivariable regression

No specific disability type showed significant associations with weekly hours in adjusted models, though communication disability (OR = 1.69, 95%CI = 1.33–2.14), restricted body movement (OR = 1.17, 95% CI = 1.01–1.35), and SpLD (OR = 1.38, 95% CI = 1.09–1.76) were associated with longer caregiving hours in univariate analyses.

### Mental Stress Experienced by Family Caregivers Due to Caregiving Responsibilities

The significant factors associated with the level of mental stress experienced by family caregivers due to caregiving responsibilities included care recipients’ age, required caregiving activities, type of disability, and caregivers’ sex (Table 3). Although care recipients in this study spanned all age groups (Table 1), the majority were aged 70 years or older. In the regression analyses, the 50–69 age group was used as the reference category to facilitate comparison with both younger and older age groups. Caring for recipients aged 70 and older was less likely to report severe mental stress compared to those aged 50–69 (aOR = 0.40, 95% CI = 0.56–0.80). In contrast, caregivers providing care for recipients requiring assistance with ADLs were more likely to experience severe mental stress (aOR = 1.99, 95% CI = 1.62–2.44). Regarding caregiver demographics, female caregivers were more likely to report severe stress compared to their male counterparts (aOR = 1.39, 95% CI = 1.15–1.69).

Regarding disability type, caregivers assisting individuals with mental illnesses or mood disorders were twice as likely to report severe stress compared to those caring for recipients without such conditions (aOR = 2.00, 95% CI = 1.64–2.43). Several other disability types including seeing disability, communication difficulties, restriction in body movement, SpLD, AD/HD, and ASD showed elevated stress levels in unadjusted analyses but were not statistically significant in the adjusted model.

## Discussion

This study shows patterns in family caregiving for individuals with disabilities in Hong Kong, a society where cultural norms and structural limitations intersect to shape caregiver burdens. Three key findings are critical to discuss: (1) the persistent influence of Confucian filial piety in gendering caregiving roles, (2) the divergent burdens associated with types of disabilities, and (3) the tension between socioeconomic resources and cultural expectations. Findings support tailored caregiver interventions based on disability and caregiver demographics.

### Main Finding of This Study

This study analyzed factors associated with caregiving provision, weekly caregiving hours, and mental stress among family caregivers for individuals with disabilities in Hong Kong. Caregiving was significantly more likely among women and unmarried individuals. Longer caregiving hours and higher stress levels were associated with assisting individuals with ADLs or those with mental health disabilities. These findings reflect the interplay of cultural norms, disability-specific demands, and socioeconomic conditions shaping caregiver burden.

### What Is Already Known on This Topic

Prior research in East Asian contexts has documented the disproportionate caregiving responsibilities placed on women, driven by Confucian norms of filial piety [3, 4]. Studies have also shown that caregiving burden can vary based on the care recipient’s condition, caregiver’s employment status, and availability of support [9–13]. However, much of this literature treats disability as a single category, overlooking how different types of disability, especially mental and developmental, uniquely affect caregiver burden.

### What This Study Adds

This study adds several key insights. First, while prior research has established that women disproportionately assume caregiving roles in East Asian contexts, our study extends this literature by demonstrating that gender differences persist across multiple dimensions of caregiving burden, including the likelihood of providing care, the intensity of care provided, and the experience of mental stress. Using population-representative data from Hong Kong, we show that female caregivers are not only more likely to take on caregiving roles, but also to provide longer hours of care and experience greater psychological strain. Importantly, these gender disparities are not uniform but vary according to caregiving contexts, particularly in situations involving high-intensity care (e.g., ADL support) and mental health–related caregiving. This highlights how gendered caregiving roles interact with disability-specific demands to shape caregiving burden. These findings underscore how cultural expectations around female caregiving persist despite increasing female labor force participation and education levels [22–24].

Second, by applying the ICF framework, we demonstrate that caregiving burdens are not uniform but vary according to disability type. Caregivers assisting individuals with ADL needs faced longer caregiving hours, likely due to the physically intensive nature of such tasks [14]. This aligns with global patterns but is exacerbated in East Asian contexts by cultural preferences for familial care over institutional solutions [25]. In multigenerational households, ADL-dependent care recipients may receive more hours of family care, as families prioritize privacy and moral obligations over external services [4]. Caregivers of individuals with mental health conditions experienced the highest levels of reported stress, consistent with literature on the emotional toll of unpredictable behaviors, stigma, and insufficient access to psychiatric support services [15, 16, 26, 27]. Although only ADL and mental health–related care emerged as significant in adjusted models, other disabilities (e.g., SpLD, AD/HD, ASD, body movement restrictions) showed significant unadjusted associations, suggesting they may still carry important burdens shaped by caregiver characteristics and available support.

Third, caregiver stress is not solely driven by the care recipient’s needs but shaped by the caregiver’s social position. Employed or more educated caregivers provided fewer hours, likely due to time constraints and greater access to formal services [28, 29]. Caregivers with additional family help reported shorter hours, reinforcing the value of shared care. However, these benefits are less accessible to women in patrilineal households, where caregiving is often viewed as their exclusive domain [30]. Gendered expectations restrict women’s ability to delegate care or access resources, particularly in mental health caregiving, where societal stigma and emotional labor compound the burden [31–33].

Interestingly, caregivers for recipients aged 70 and older were less likely to report stress, diverging from studies suggesting higher burden for older care recipients [34, 35]. This may reflect more predictable routines or better access to eldercare resources in Hong Kong [36, 37].

A key strength of this study lies in its focus on an often-overlooked group: family caregivers of individuals with disabilities in East Asian societies. While policy efforts in the region have largely centered on eldercare, support systems for disability-related caregiving remain fragmented or absent. By drawing attention to this policy blind spot, our study offers timely insights for improving caregiver support in contexts where familial care is culturally expected but structurally unsupported. Our analysis also benefits from the use of population-representative data from the Hong Kong General Household Survey (*n* = 40,600). This large, high-quality dataset allows us to assess caregiving dynamics in a high-density, non-Western urban environment where Confucian values, aging populations, and limited long-term care infrastructure intersect. Finally, by examining both caregiving provision and intensity, this study captures the full range of caregiver burden. Such analysis is essential for developing interventions that address not only the quantity of care but also the quality of support. These contributions advance the field by demonstrating that disability heterogeneity matters, particularly in aging societies grappling with rising disability prevalence.

### Policy and Practice Implications

Our findings highlight several actionable policy priorities for Hong Kong. First, establish gender-sensitive caregiver support that reduces the opportunity costs borne by women. Across OECD countries, common measures include direct and indirect cash benefits and care-leave entitlements, reflecting recognition that intensive informal care can constrain labor market participation and harm mental health [38, 39]. In Hong Kong, recent measures such as regularized living allowances for low-income carers and the territory-wide carer support agenda provide an institutional basis to further tailor support to working-age women caregivers, including flexible work arrangements encouraged through employer guidance and targeted financial relief for high-burden carers [37].

Second, expand respite and formal home-based services for caregivers managing high-intensity ADL assistance. Existing Social Welfare Department home-based programs already include home respite and carer support components [40]. Scaling service capacity and improving access could directly address the time burden identified in our models. Internationally, Japan’s Long-Term Care Insurance (LTCI) system illustrates an integrated approach in which care recipients undergo standardized needs assessment and receive coordinated packages of home help, day care, and short-stay respite services within a unified framework [40]. Adapting this model to Hong Kong would involve strengthening care coordination (e.g., case managers), improving integration between disability and eldercare services, and streamlining access pathways.

Third, integrate mental health support into caregiver pathways. Hong Kong’s 24-h carer hotline provides counselling, crisis support, and referral, which could be complemented by brief evidence-informed caregiver training and peer support [41]. The World Health Organization iSupport model demonstrates how scalable skills-and-coping programs can be delivered online or offline, and could be adapted (e.g., Cantonese materials) for caregivers experiencing stress [42].

Fourth, provide targeted support for caregiving involving mental health–related disabilities. WHO guidance emphasizes community-based, rights-oriented mental health services and peer support. Embedding caregiver psychoeducation, crisis planning and stigma-sensitive support within community mental health and rehabilitation services could reduce the emotional labor and uncertainty reported in such care contexts [43]. Importantly, in Hong Kong, in-home support is often provided by both family members and co-residing caregivers, including foreign domestic workers and privately hired helpers. As these groups constitute a substantial component of caregiving support, respite services, training programs, and caregiver policies should consider “family members and co-residing caregivers” as co-beneficiaries.

### Limitations of This Study

Several limitations must be acknowledged. Firstly, the mental stress status of caregivers was self-reported, rather than measured using standardized screening tools such as the PHQ-9 for depression or the GAD-7 for anxiety. Additionally, 13.3% of the responses in the mental stress variable reported “did not know” or refusing to answer. These factors could cause reporting bias, reduce the accuracy of the findings, and limit the ability to generalize results. Thirdly, given the cross-sectional nature of the study, causality cannot be inferred. There was no longitudinal follow-up to determine whether observed associations between caregiving factors and burdens evolve over time. Therefore, future studies employing longitudinal designs would help clarify causal relationships. Finally, the study did not account for other potentially relevant variables, such as household income and household size due to the lack of data, which may influence caregiving capacity and burden. Including these variables in future research could yield a more detailed understanding of the social and economic context of caregiving.

## Conclusion

This study examined how individual, contextual, and disability-specific factors influence caregiving provision, time intensity, and mental stress among family caregivers for individuals with disabilities in Hong Kong. Female caregivers, especially those supporting individuals with ADL needs or mental health disabilities, experienced the highest time burden and psychological stress. Findings call for targeted, culturally sensitive caregiver policies that reflect gender and care need diversity. Public health systems in aging, high-density East Asian societies where informal caregiving is prevalent must recognize this diversity to design sustainable and equitable caregiver support policies.

## Data Availability

The data underlying this article were provided by Hong Kong Census & Statistics Department (C&SD) by permission. Data will be available upon reasonable request by contacting the Hong Kong Census & Statistics Department (C&SD) and obtaining the necessary permissions.

## Appendix

Table 4.

**Table 4:** Logistic regression analysis of associations between care recipients’ needs, family caregivers’ characteristics, contextual factor, and weekly caregiving hours provided by caregivers and mental stress severity experience by family caregiver due to caregiving responsibilities for people with disabilities

Variables	Weekly caregiving hours provided		Level of mental stress felt	
	Crude OR (95% CI)	Adjusted OR (95% CI)^†^	Crude OR (95% CI)	Adjusted OR (95% CI)^†^
***Care recipient***				
**Sex of care recipient (ref: female)**				
Male	1.48 (1.28, 1.71)	0.90 (0.75, 1.07)	1.48 (1.26, 1.74)	1.19 (0.97, 1.46)
**Age of care recipient (ref: 50–69)**				
< 15	1.32 (1.03, 1.69)	2.15 (1.15, 4.04)	1.69 (1.27, 2.24)	2.03 (1.00, 4.11)
15–29	1.13 (0.81, 1.58)	1.91 (1.10, 3.29)	1.66 (1.13, 2.42)	1.57 (0.84, 2.92)
30–49	0.89 (0.64, 1.24)	1.09 (0.72, 1.64)	1.19 (0.82, 1.72)	1.09 (0.68, 1.74)
> = 70	1.02 (0.84, 1.25)	0.85 (0.67, 1.09)	0.73 (0.58, 0.91)	0.60 (0.56, 0.80)**
**Caring activity required**				
ADLs (ref: do not need ADLs)	2.00 (1.73, 2.32)	2.54 (2.12, 3.04)***	1.54 (1.31, 1.81)	1.99 (1.62, 2.44)***
IADLs (ref: do not need IADLs)	0.65 (0.52, 0.81)	0.49 (0.32, 0.76)	0.54 (0.42, 0.69)	0.86 (0.54, 1.36)
**Type of disability**				
Seeing disability (ref: no seeing disability)	0.84 (0.67, 1.06)	0.91 (0.72, 1.16)	0.69 (0.52, 0.90)	0.83 (0.62, 1.12)
Hearing disability (ref: no hearing disability)	0.95 (0.75, 1.20)	-	0.95 (0.73, 1.23)	-
Communication disability (ref: no communication disability)	1.69 (1.33, 2.14)	1.34 (1.04, 1.72)	1.64 (1.27, 2.11)	1.21 (0.92, 1.59)
Restriction in body movement (ref: no restriction)	1.17 (1.01, 1.35)	1.09 (0.89, 1.35)	0.81 (0.69, 0.95)	1.39 (1.07, 1.80)
SpLD (ref: no SpLD)	1.38 (1.09, 1.76)	1.14 (0.85, 1.53)	1.62 (1.24, 2.12)	0.83 (0.60, 1.15)
AD/HD (ref: no AD/HD)	1.08 (0.86, 1.35)	-	2.12 (1.63, 2.76)	1.24 (0.87, 1.76)
ASD (ref: no ASD)	1.07 (0.83, 1.38)	-	1.92 (1.45, 2.54)	1.06 (0.74, 1.51)
Mental illness/mood disorder (ref: no mental illness)	1.02 (0.87, 1.19)	-	1.72 (1.45, 2.05)	2.00 (1.64, 2.43)***
***Family caregiver***				
**Sex (ref: female)**				
Male	0.58 (0.50, 0.67)	0.75 (0.63, 0.89)**	0.60 (0.51, 0.71)	0.72 (0.59, 0.87)**
**Employment status of caregiver (ref: unemployed)**				
Employed	0.25 (0.21, 0.29)	0.35 (0.29, 0.41)***	0.83 (0.71, 0.98)	0.97 (0.80, 1.17)
**Marital status of caregiver (ref: currently married)**				
Never married	0.52 (0.44, 0.61)	0.93 (0.73, 1.18)	0.74 (0.62, 0.89)	1.01 (0.78, 1.31)
Widowed/divorced/separated	0.84 (0.66, 1.09)	0.85 (0.65, 1.13)	1.39 (1.06, 1.82)	1.26 (0.93, 1.70)
**Educational attainment of caregiver (ref****: ****lower/upper secondary)**				
Below primary	1.60 (1.34, 1.92)	1.16 (0.94, 1.43)	0.88 (0.72, 1.09)	0.96 (0.75, 1.23)
Post-secondary	0.45 (0.37, 0.53)	0.62 (0.51, 0.75)***	0.80 (0.66, 0.97)	0.88 (0.71, 1.09)
***Contextual***				
**If have extra help (ref: no)**				
Yes	0.54 (0.47, 0.63)	0.55 (0.47, 0.64)***	0.94 (0.80, 1.11)	-

****p* <.001, ***p* <.01, **p* <.05, *p*-values are adjusted for multiple comparisons using the Benjamini-Hochberg (FDR) procedure; *n* = 148,000

^**†**^Adjusted odds ratios (aOR) are derived from multivariate ordinal logistic regression analyses, where only variables with a *p*-value < 0.25 in univariate analysis were included in the multivariate model

“-” indicates variables not entered into the multivariable model. Variables with *p* ≥ 0.25 in univariate analyses were not included in the multivariable regression

Abbreviations: *AD/HD*, attention deficit/hyperactivity disorder; *ADLs*, activities of daily living; *ASD*, autism spectrum disorder; *IADLs*, instrumental activities of daily living; *SpLD*, specific learning difficulties
